# Image-guided Percutaneous Polymethylmethacrylate-augmented Spondylodesis for Painful Metastasis in the Veteran Population

**DOI:** 10.7759/cureus.4509

**Published:** 2019-04-20

**Authors:** Eric S Sussman, Allen Ho, Arjun V Pendharkar, Suzanne Tharin

**Affiliations:** 1 Neurosurgery, Stanford University School of Medicine, Stanford, USA

**Keywords:** spondylodesis, minimal access spine surgery, cement-augmentation, polymethylmethacrylate, spinal metastases

## Abstract

The treatment of painful spinal metastases in patients with limited life-expectancy, significant perioperative risks, and poor bone quality poses a surgical challenge. Recent advances in minimal-access spine surgery allow for the surgical treatment of patients previously considered not to be operative candidates. The addition of fenestrated screws for cement augmentation to existing image-guided percutaneous pedicle screw fixation can enhance efficiency, decrease risk of hardware complications, and improve back pain in this patient population.

The patient is a 70-year-old man with severe axial back pain due to metastatic prostate cancer and L5 pathologic fractures not amenable to kyphoplasty. In the setting of a 6-12-month life-expectancy, the primary goal of surgery was relief of back pain associated with instability with minimal operative morbidity and post-operative recovery time. This was achieved with an internal fixation construct including percutaneously placed cement-augmented fenestrated pedicle screws at L4 and S1. The patient was discharged to home on post-operative day 1 with substantial improvement of his low back pain.

Image-guided, percutaneous placement of fenestrated, cement-augmented pedicle screws is an emerging treatment for back pain associated with metastasis. Fenestrated screws allow for integrated cement augmentation. The minimal associated blood loss and recovery time make this approach an option even for patients with limited life-expectancy. This is the first report of utilization of this technique for the veteran population.

## Introduction

Within the veteran population, the prevalence of cancer and development of pain and debilitating spinal metastasis has become a frequently encountered challenge for spine surgeons. The rate of spine metastasis causing some degree of spinal cord compression in cancer patients is about five percent [1-2]. In the veteran population, 43% of patients with cancer and back pain were found to have spinal metastases [3]. The development of percutaneous techniques allows for spinal instrumentation in a cohort of patients that were previously not surgical candidates. However, instrumentation without arthrodesis, particularly in boor quality bone, carries the risk of hardware failure [4]. Augmentation of spinal hardware with polymethylmethacrylate (PMMA) has been shown to reduce the incidence of screw pullout in osteoporotic patients [5-6]. Cannulated and fenestrated screws have allowed for the improved safety and convenience of this technique [7], and polyaxial fenestrated screws have been subsequently developed to facilitate PMMA-augmentation of percutaneously placed hardware [8-10]. This has proved to be especially an effective tool for the treatment of instability-related pain secondary to spinal metastasis, minimizing morbidity and recovery time in patients where quality of life is the primary goal [11]. Percutaneous PMMA augmentation has been reported for this same indication with good clinical results, though this was completed without fenestrated screws in the nonveteran population [11]. The advent of fenestrated screws amenable to percutaneous image-guided placement represents a technical advance to streamline the operation. Here, we report the first use of PMMA-augmentation via image-guided, percutaneously placed fenestrated pedicle screws in a veteran patient for the treatment of axial bone pain from lumbar spinal metastasis.

## Case presentation

**Figure 1:**
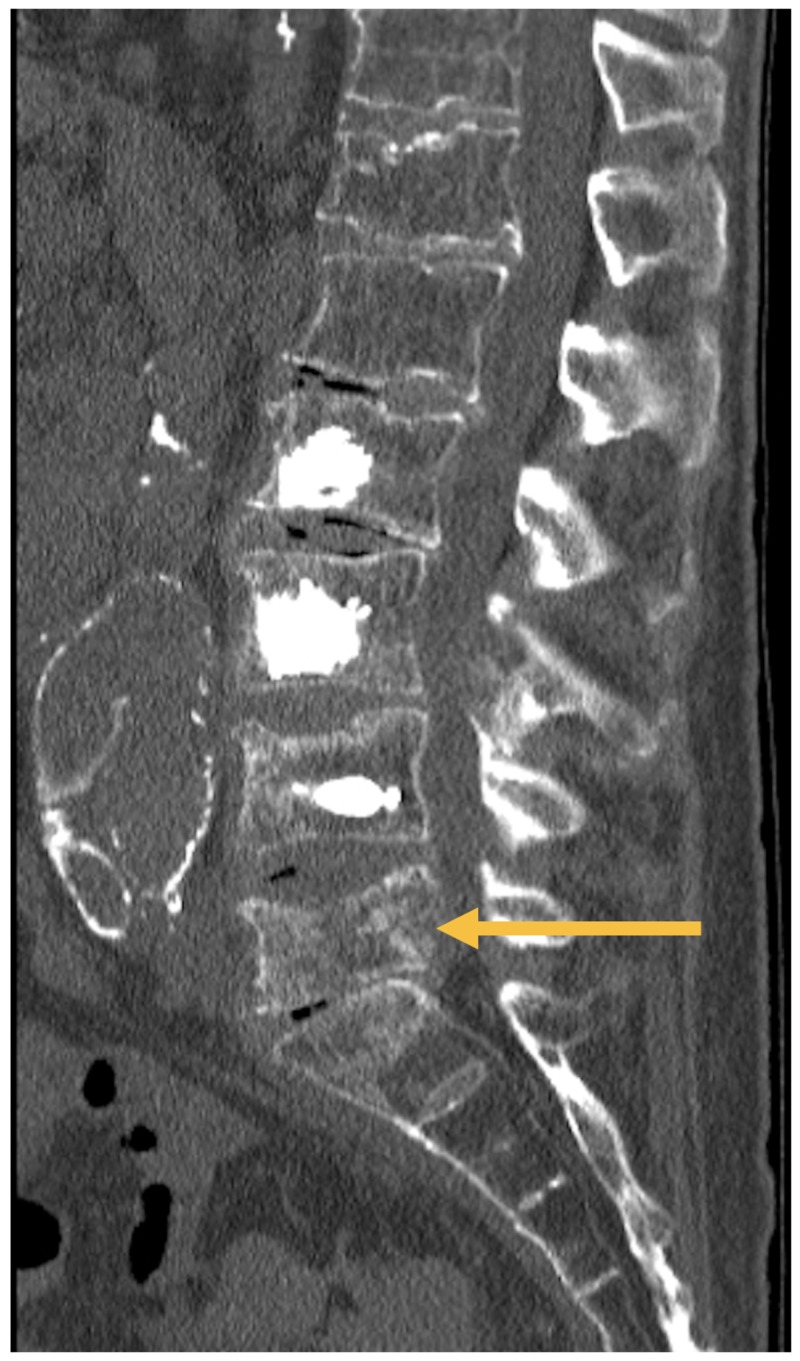
Lateral plain films of the lumbar spine, demonstrating a compression fracture of the L5 vertebral body, with approximately 50% loss of height and mild retropulsion into the spinal canal, and disruption of the posterior endplate. There is also radiographic evidence of prior cement augmentation of the L2, L3, and L4 vertebral bodies.

The patient is a 70-year-old male veteran with a history of metastatic prostate cancer and diffuse metastases throughout his spine, including large vertebral metastases from L2-L5 with resultant pathologic fractures at these levels. His life-expectancy was estimated to be 6-12 months. He presented with severe axial low back and groin pain. He notably denied lower extremity radiculopathy or symptoms of neurogenic claudication, and his sensorimotor exam was normal. He was initially treated with radiation therapy, which provided relief of his groin pain without any significant effect on his low back pain. He subsequently underwent vertebroplasty to L2, L3, and L4 with partial relief of symptoms. The L5 vertebral body was not a suitable target for vertebroplasty, as the fracture at that level resulted in violation of the posterior vertebral body wall (Figure 1). He continued to have load- and movement-dependent low back pain that limited his mobility, required opiates to control, and negatively impacted his quality of life. Lumbrosacral orthosis mildly but incompletely improved his pain.

In the context of persistent low back pain and the contraindication to vertebroplasty of an L5 pathologic fracture, it was felt that the patient would benefit from L4-S1 fixation. The primary goal of this operation was to provide symptomatic relief of axial low back pain while minimizing operative morbidity and recovery time. Thus, the patient elected to undergo image-guided percutaneous pedicle screw instrumentation and internal fixation. Due to the co-existence of osteoporosis and overall poor bone quality secondary to diffuse spinal metastases, the decision was made to perform PMMA-augmentation of the fusion construct.

Percutaneous pedicle screw placement at L4 and S1 was performed with the assistance of an O-arm and a StealthStation S7 Surgical Navigation System (Medtronic, Inc. Minneapolis, MN. USA). A percutaneous image-guidance reference pin was placed into the right iliac crest, and the O-arm was subsequently used to obtain images for navigation. Using the Stealth Navigation System, starting points for the L4 screws were identified, and stab incisions were made approximately 4.5 cm lateral to the midline. Using a guidewire-based technique, 6.5 mm x 45 mm CD Horizon Solera fenestrated pedicle screws (Medtronic, Inc.) were placed into the bilateral L4 pedicles. Using the same operative technique, 6.5 mm x 45 mm fenestrated Solera pedicle screws were placed into the bilateral S1 pedicles.

**Figure 2:**
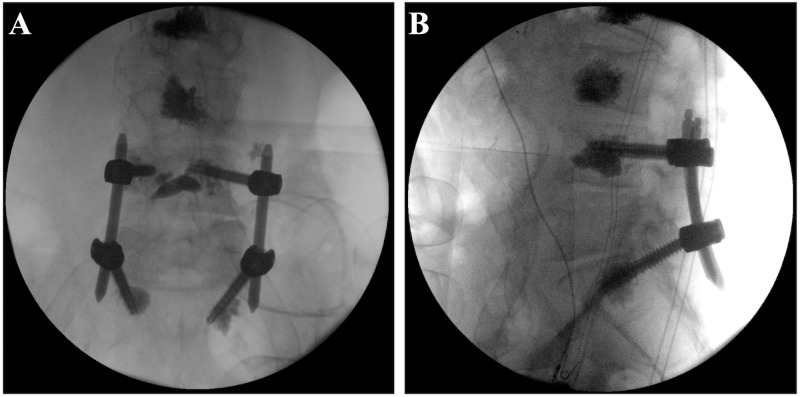
Intraoperative AP (A) and lateral (B) plain films of the lumbosacral spine, demonstrating optimally placed bilateral L4 and S1 PMMA-augmented pedicle screws.

Driver shafts were then re-threaded into each of the screw tulip heads. A kyphoplasty needle was inserted into each of the driver shafts and 1.5 cc of Kyphon Xpede (PMMA) Bone Cement (Medtronic, Inc.) was injected through each fenestrated pedicle screws under lateral fluoroscopic guidance. A 3.5 mm x 90 mm lordotic rod was then passed between the tulip heads of the pedicle screws via the L4 incision, reduced into the tulip heads, and secured with set screws. Final antero-posterior and lateral X-rays confirmed optimal placement of this percutaneously placed, PMMA-augmented fusion construct (Figure 2).

**Figure 3:**
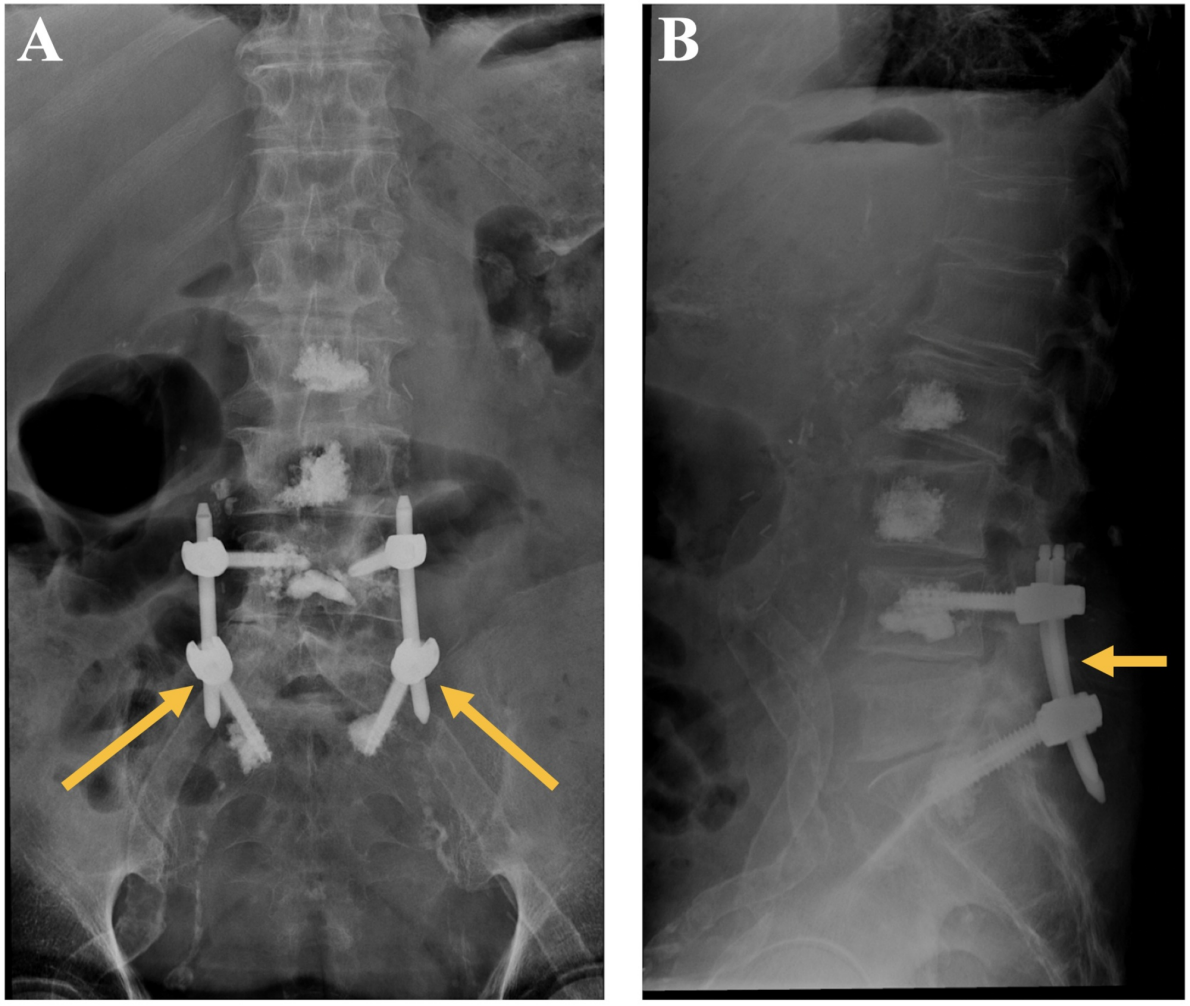
Repeat AP (A) and lateral (B) plain films of the lumbosacral spine two weeks post-operatively, demonstrating intact L4-S1 instrumentation, and early evidence of posterolateral bony fusion mass.

On post-operative day one, the patient’s axial low back pain had improved substantially, his sensorimotor exam remained intact, and he was discharged to home. Plain films of the lumbosacral spine obtained at two-week follow up revealed an intact lumbosacral instrumented construct (Figure 3).

## Discussion

This is the first report of an image-guided, percutaneous, PMMA-augmented, lumbosacral instrumented fixation for treatment of back pain from metastasis to the spine in the veteran population. The primary objective of this operation was to treat the patient’s pain while minimizing operative morbidity and recovery time. Importantly, this patient was at high risk of hardware failure due to the presence of diffuse vertebral metastases, pathologic fractures and osteoporosis, which was further exacerbated by prior radiation therapy. Thus, screw augmentation with PMMA bone cement was employed to maximize the strength and durability of the construct.

Cement augmentation of percutaneous screw fixation of unstable spinal metastasis has been demonstrated to be a safe and effective two-step procedure [11]. The addition of fenestrated screws can further streamline this operation. Fenestrated screws for cement augmentation have been in use in Europe and represent a recent innovation in North America [10]. Biomechanical studies have demonstrated that fenestrated screws have increased pullout strength with PMMA augmentation compared to conventional screw and cement augmentation constructs [12]. Recent data suggest reduced life-expectancy in prostate cancer in the veteran compared to the nonveteran cohort [13]. Given the short life-expectancy associated with widely metastatic prostate and other cancers, often open surgical fusion for relief of mechanical pain from pathologic fractures is forgone due to concerns about prolonged post-operative recovery times [14]. Based on the landmark study by Patchell et al. [15], a minimum of three months of life-expectancy is widely considered necessary to derive benefit from a spinal decompression and stabilization procedure [16-18]. However, as demonstrated in this report, navigated, percutaneously placed, fenestrated pedicle screws with cement augmentation and internal fixation may represent a low morbidity, rapid recovery procedure to add to the surgeon’s armamentarium to provide significant stabilization and pain relief to patients in this population previously not considered to be surgical candidates. This technique raises the possibility of reducing pain and narcotic use even in a patient with a two to three month life-expectancy.

## Conclusions

Image-guided percutaneous cement-augmented fenestrated pedicle screw placement is an emerging operative technique for spinal stabilization and pain relief for axial back pain in the setting of metastatic disease of the spine. In the appropriately selected patient, this technique may provide stable constructs and pain relief with minimal operative morbidity and recovery time.
